# Big data analytics for MerTK genomics reveals its double-edged sword functions in human diseases

**DOI:** 10.1016/j.redox.2024.103061

**Published:** 2024-02-05

**Authors:** Shijie Liu, Jinzi Wu, Daixuan Yang, Jianliang Xu, Hang Shi, Bingzhong Xue, Zufeng Ding

**Affiliations:** Department of Biology, Georgia State University, Atlanta, GA, 30303, USA

**Keywords:** Big data analytics, RNA-seq, MerTK, Cardiovascular disease, Immunity

## Abstract

**Rationale:**

MER proto-oncogene tyrosine kinase (MerTK) is a key receptor for the clearance of apoptotic cells (efferocytosis) and plays important roles in redox-related human diseases. We will explore MerTK biology in human cells, tissues, and diseases based on big data analytics.

**Methods:**

The human RNA-seq and scRNA-seq data about 42,700 samples were from NCBI Gene Expression Omnibus and analyzed by QIAGEN Ingenuity Pathway Analysis (IPA) with about 170,000 crossover analysis. MerTK expression was quantified as Log2 (FPKM + 0.1).

**Results:**

We found that, in human cells, MerTK is highly expressed in macrophages, monocytes, progenitor cells, alpha-beta T cells, plasma B cells, myeloid cells, and endothelial cells (ECs). In human tissues, MerTK has higher expression in plaque, blood vessels, heart, liver, sensory system, artificial tissue, bone, adrenal gland, central nervous system (CNS), and connective tissue. Compared to normal conditions, MerTK expression in related tissues is altered in many human diseases, including cardiovascular diseases, cancer, and brain disorders. Interestingly, MerTK expression also shows sex differences in many tissues, indicating that MerTK may have different impact on male and female. Finally, based on our proteomics from primary human aortic ECs, we validated the functions of MerTK in several human diseases, such as cancer, aging, kidney failure and heart failure.

**Conclusions:**

Our big data analytics suggest that MerTK may be a promising therapeutic target, but how it should be modulated depends on the disease types and sex differences. For example, MerTK inhibition emerges as a new strategy for cancer therapy due to it counteracts effect on anti-tumor immunity, while MerTK restoration represents a promising treatment for atherosclerosis and myocardial infarction as MerTK is cleaved in these disease conditions.

## Highlights

• MerTK is a key receptor for efferocytosis that is a process for the clearance of apoptotic cells. MerTK plays an important role in redox homeostasis.
• Big data analytics for MerTK biology are based on bulk RNA-seq, scRNA-seq data, and BioGPS.
• MerTK has completely different functions depending on disease types and sex differences.
• The specific pharmacologic approaches to regulate MerTK expression are promising clinical strategies that are worthy for future investigation.

## Graphical abstract

Characteristics of MerTK receptor in human cells, tissues, and diseases. MerTK is highly expressed in immune cells, vascular cells, and stem cells, affecting the pathological process of the three most common diseases in humans, such as brain disorders, cardiovascular diseases, and cancer. MerTK expression also shows sex differences in a variety of human diseases. This figure was created with BioRender.com.

**Figure:**
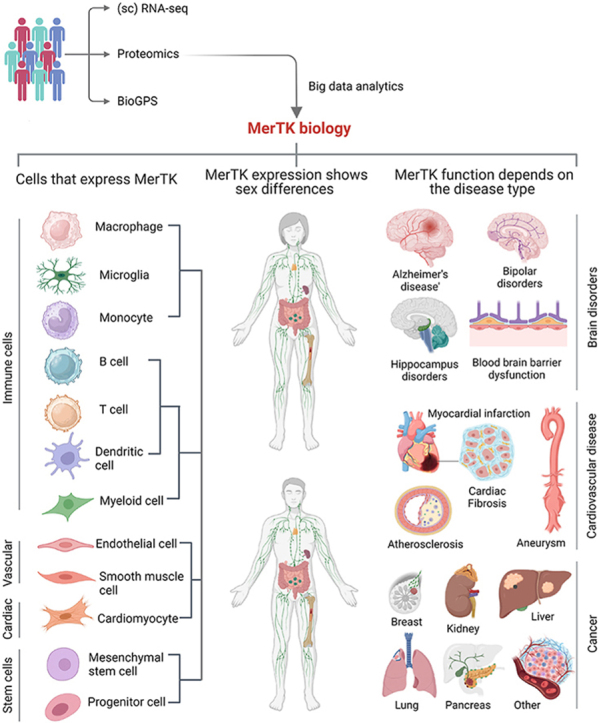

## 1 Introduction

MER proto-oncogene tyrosine kinase (MerTK), a member of the TAM (Tyro3, Axl and MerTK) receptor family, plays important roles in redox homeostasis and accelerates the efficient clearance of apoptotic cells, a process called efferocytosis [1]. Efficient efferocytosis promotes differentiation of phagocytes into an anti-inflammatory phenotype that attenuates oxidative damage, whereas impaired efferocytosis leads to the detrimental accumulation of apoptotic cells and increases levels of free radicals. In the cardiovascular system, for example, MerTK cleavage promotes impaired efferocytosis and defective inflammation resolution, leading to atherosclerotic plaque necrosis [2,3]. MerTK is mainly expressed on the surface of macrophages and dendritic cells [4] while lower expressed in vascular endothelial cells (ECs) [5] and vascular smooth muscle cells (SMCs) [6]. It also is physiologically expressed in other cell types including microglial, monocytes, natural killer (NK) cells, epithelial cells, and platelets [7]. Although MerTK is absent from normal B and T lymphocytes, it is highly expressed in activated B and T lymphocytes in the tumor microenvironment [[8], [9]]. MerTK also is highly expressed in the central nervous system (CNS), lung, kidney, prostate cancer, and diseased tissues including metastatic prostate cancer and liver fibrosis [2,3,10]. MerTK is expressed at low levels in aorta, heart, and skeletal muscle [5,11,12].

MerTK is implicated in a wide variety of diseases including cancer, cardiovascular disease and brain pathologies [2,3]. An increase in its abundance during a disease state may represent a compensatory response to enact tissue protection and retention of function, whereas a deficiency of MerTK has been linked to worsened outcomes [2,3]. Accordingly, MerTK is overexpressed as an abnormal finding in many human cancers including melanoma, leukemia, and prostate, lung, breast and liver cancer [13]. MerTK also is considered a potentially novel therapeutic target for the treatment of cancer since it can regulate the innate immune response, tissue homeostasis and repair, and platelet aggregation [7]. In brain, MerTK is mainly expressed in microglia, infiltrated macrophages and astrocytes [14], and its expression increases in brain disorders such as traumatic brain injury (TBI), stroke and brain tumors [15,16]. Conversely, MerTK is cleaved in advanced atherosclerosis and MerTK deficiency is associated with increased infarct size and cardiac dysfunction [3,11]. Mice with resistant cleavage of MerTK consistently show reduced infarct size and improved cardiac function [11].

Here, we relied on big data analytics based on RNA-seq or scRNA-seq combined with mRNA expression profiles available from BioGPS (http://biogps.org) to provide a summary of MerTK gene expression in normal and diseased human cells and tissues. We report broad abnormalities of MerTK expression in an array of human diseases including inflammation, cancer, diabetes, kidney failure, hepatitis, and diseases of the brain, cardiovascular system and thyroid in addition to aging. We also detail sex differences in tissue specific MerTK expression. Our big data analytics concludes with a commentary on implementing MerTK inhibition or restoration as new therapeutic strategies to mitigate human diseases, especially those resistant to current treatments, and the challenges that may be associated with these interventions. Our data are intended to provide cues to investigators interested in MerTK biology across a range of human systems related to abnormalities in MerTK expression that may potentially contribute to different pathologies.

## 2 Methods

**Large data analytics.** The mRNA expression data were from BioGPS (http://biogps.org). The RNA-seq or scRNA-seq data were downloaded from QIAGEN Ingenuity Pathway Analysis and QIAGEN OmicSoft Land Explorer [17], with about 42,700 samples for about 170,000 crossover analysis of human cells, tissues, and diseases. Fragments per kilo base of transcript per million mapped fragments (FPKM) is an expression level normalization method as a gene expression unit in RNA-seq. Log2 (FPKM + 0.1) was used to quantify MerTK expression. The data were further sorted by cell types, tissue types with different diseases, and sex differences. GraphPad Prism 9.4.1. was used to analyze, graph, and present the BioGPS and RNA-seq data. Based on https://qiagen.my.salesforcesites.com/KnowledgeBase/KnowledgeNavigatorPage?id=kA41i000000L5pXCAS&categoryName=IPA, a data set containing gene identifiers and corresponding data measurement values was uploaded into the application. Each identifier was mapped to its corresponding entity in QIAGEN's Knowledge Base. Network Eligible molecules were overlaid onto a global molecular network developed from information contained in the QIAGEN Knowledge Base. Networks of Network Eligible Molecules were then algorithmically generated based on their connectivity. The Diseases & Functions Analysis identified the biological functions and/or diseases that were most significant from the data set. Molecules from the dataset that were associated with biological functions and/or diseases in the QIAGEN Knowledge Base were considered for the analysis. A right-tailed Fisher's Exact Test was used to calculate a p-value determining the probability that each biological function and/or disease assigned to that data set is due to chance alone. A z-score was calculated to indicate the likelihood of increase or decrease of that disease or function. There are about 1500 disease, phenotype, and function pathways was created by machine learning (ML) in the QIAGEN Knowledge Base. These ML Disease Pathways show key molecules that impact a single disease and its associated phenotypes, which may represent novel participants in the disease or its etiology.

**Animals.** Male wildtype mice on the C57BL/6 background were purchased from Jackson Laboratories (Sacramento, CA, USA) and housed in the Division of Laboratory Animal Medicine at our institution. All experimental procedures were performed in accordance with protocols approved by the Institutional Animal Care and Use Committee and conformed to the Guidelines for the Care and Use of Laboratory Animals published by the US National Institutes of Health. After mice were euthanized by CO_2_ asphyxiation, the aortic arch was carefully dissected from surrounding tissue, then fixed with 10 % neutral buffered formalin solution (Sigma, HT501128), and embedded in paraffin for further immunohistochemical analyses.

**Cells.** Primary human aortic ECs (HAECs) and the human Jurkat cell line were purchased from ATCC (Manassas, VA, USA). HAECs were cultured with Vascular Cell Basal Medium (ATCC, PCS-100-030) and Endothelial Cell Growth Kit (ATCC, PCS-100-041). Jurkat cells were cultured in RPMI-1640 medium (ATCC, 30–2001) with 10 % Fetal Bovine Serum (FBS, ATCC 30–2020).

**EC efferocytosis measurement.** EC efferocytosis was analyzed by a modified protocol [2]. Briefly, ECs were labeled with PKH26 Red Fluorescent Cell Linker Kit (Sigma), seeded on a 6-well cell culture plate (1 × 10^6^ cells/well), and allowed to reach confluence. Jurkat cells were labeled with PKH67 Green Fluorescent Cell Linker Kit (Sigma) and exposed to UV light (254 nm, UVP) for 5 min to induce apoptosis. The apoptotic Jurkat cells were incubated at 37 °C with 5 % CO_2_ for 1 h. EC medium was replaced with medium containing apoptotic Jurkat cells to achieve a cell ratio of 3:1, or as indicated for apoptotic Jurkat cells/ECs. After incubation for 1 h at 37 °C, the ECs were washed twice with cold PBS. The percentage of ECs labeled with PKH67-GL from engulfing apoptotic cells were quantified with a fluorescence microscopy.

**Western blotting.** Protein was extracted with RIPA Lysis Buffer System (Santa Cruz, CA, USA) and loaded onto Mini-PROTEAN® TGX™ Precast Gels (Bio-rad, CA, USA) for electrophoresis. The size-separated proteins were then transferred to Hybond ECL Nitrocellulose Membranes (GE Healthcare, NJ, USA). After blocking with 5 % BSA buffer for 1 h, the membranes were incubated with primary antibody recognizing MerTK (Abcam, ab95925) or β-actin (Abcam, ab227387) at 1:1000 dilution overnight at 4 °C. After washing with PBS containing 0.1 % Tween-20, membranes were incubated with secondary antibody targeting either anti-rabbit (Abcam, ab6721) or anti-mouse (Abcam, ab6708) at 1:4000 dilution for 1 h and signals were detected with Pierce ECL western blotting substrate (Thermo Fisher Scientific, MA, USA). Intensity quantification of the bands was performed with Image J software and normalized to β-actin.

**Immunohistochemical staining.** For immunohistochemical analyses, 5 μm thick sections of the aorta were stained with indicated antibodies and analyzed using Mouse/Rabbit Specific HRP/3,3′-diaminobenzidine detection immunohistochemistry kit (Abcam, ab64264) according to the provided protocol.

**Proteomics analysis in HAECs**. HAECs were transfected with MerTK CRISPR/Cas9 KO Plasmid (Santa Cruz, sc-421631) or control plasmid with UltraCruz® Transfection Reagent (Santa Cruz, sc-395739) according to the provided protocol. Proteomics were performed by the IDeA National Resource for Quantitative Proteomics Core at University of Arkansas for Medical Sciences (UAMS). Proteins with an FDR adjusted p-value <0.05 and a fold change >2 were considered significant. The proteomics data were analyzed by QIAGEN Ingenuity Pathway Analysis (IPA) software.

**Statistical analysis.** An unpaired Student's t-test was used to determine statistical significance between two groups. Dunnett's one-way ANOVA was used for multiple comparisons between disease types and normal control. Data were analyzed with GraphPad Prism 9.4.1 and summarized as the mean ± SD. P < 0.05 was considered statistically significant.

## 3 Results

### 3.1 MerTK expression in human cells

**Fig. 1:**
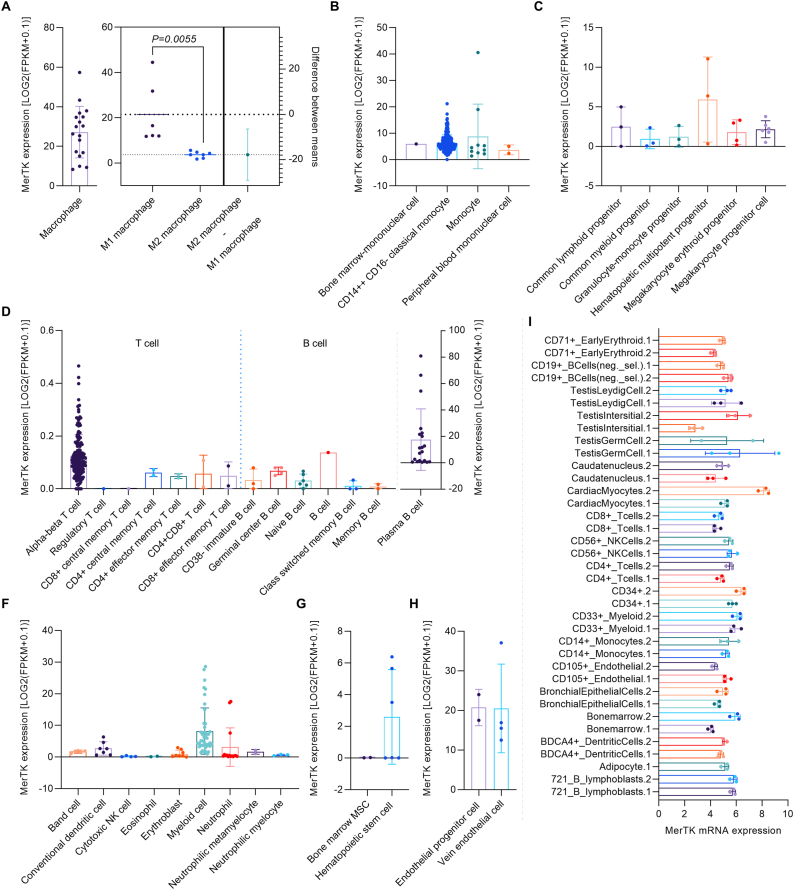
**MerTK expression in human cells.** (**A-H**) RNA-seq or scRNA-seq for MerTK expression in a variety of human cells as indicated. (**I**) MerTK mRNA expression in human cells from BioGPS. Original data of RNA-seq or scRNA-seq for MerTK expression, quantified by Log2 (FPKM + 0.1), were downloaded from QIAGEN OmicSoft Land Explorer. The data were analyzed with GraphPad Prism 9.4.1 and shown as the mean ± SD. An unpaired *t*-test was used to compare the mean two groups. P < 0.05 was considered statistically significant.

MerTK is the major receptor used by macrophages to efficiently clear apoptotic cells [2,3]. There are two phenotypically distinct subpopulations of macrophages entitled M1 and M2 [18]. M1 macrophages exhibit a pro-inflammatory phenotype and serve the function of initiating an immune response [18]. In contrast, M2 macrophages exhibit an anti-inflammatory phenotype that facilitates wound healing and tissue repair. Our analysis showed that MerTK is highly expressed in normal human macrophages isolated from cord or peripheral blood, and its expression in M1 macrophages is much higher compared to M2 macrophages (Fig. 1A). In other mononuclear cells, MerTK expression was detected in bone marrow derived mononuclear cells, CD14^++^CD16^−^ classical monocytes, common monocytes, and peripheral blood mononuclear cells (Fig. 1B). Progenitor cells can differentiate into a specific cell type and have the abilities of self-renewal, proliferation, and tissue repair [19]. Interestingly, a variety of progenitors express MerTK, inferring a potentially novel role for MerTK in the regulation of progenitor cell function (Fig. 1C).

The coordination between T and B cell lymphocytes plays a central role in the adaptive immune response [20]. In this regard, we found that MerTK is highly expressed in activated plasma B cells under the tumor microenvironment (Fig. 1D), which have an expression level similar to macrophages (Fig. 1A). In contrast, MerTK expression is very low in other T cells and B cells (Fig. 1D). Analysis of other human cells revealed that MerTK is highly expressed in myeloid cells, neutrophil and conventional dendritic cells (Fig. 1F). In stem cells, MerTK is highly expressed in hematopoietic stem cells and has detectable expression in bone marrow mesenchymal stem cells (MSC) (Fig. 1G). Recently we found that MerTK is highly expressed in primary human aortic ECs and plays important roles in vascular aging [5]. Consistently, RNA-seq data showed that MerTK has high expression in both endothelial progenitor cells and vein ECs (Fig. 1H). To confirm RNA-seq data from human cells, MerTK transcript expression was analyzed by BioGPS (Fig. 1I). As expected, MerTK is highly expressed in bone marrow derived cells, monocytes, T cells, B cells, endothelial cells, and cardiac myocytes (Fig. 1I).

### 3.2 MerTK expression in human tissues

**Fig. 2:**
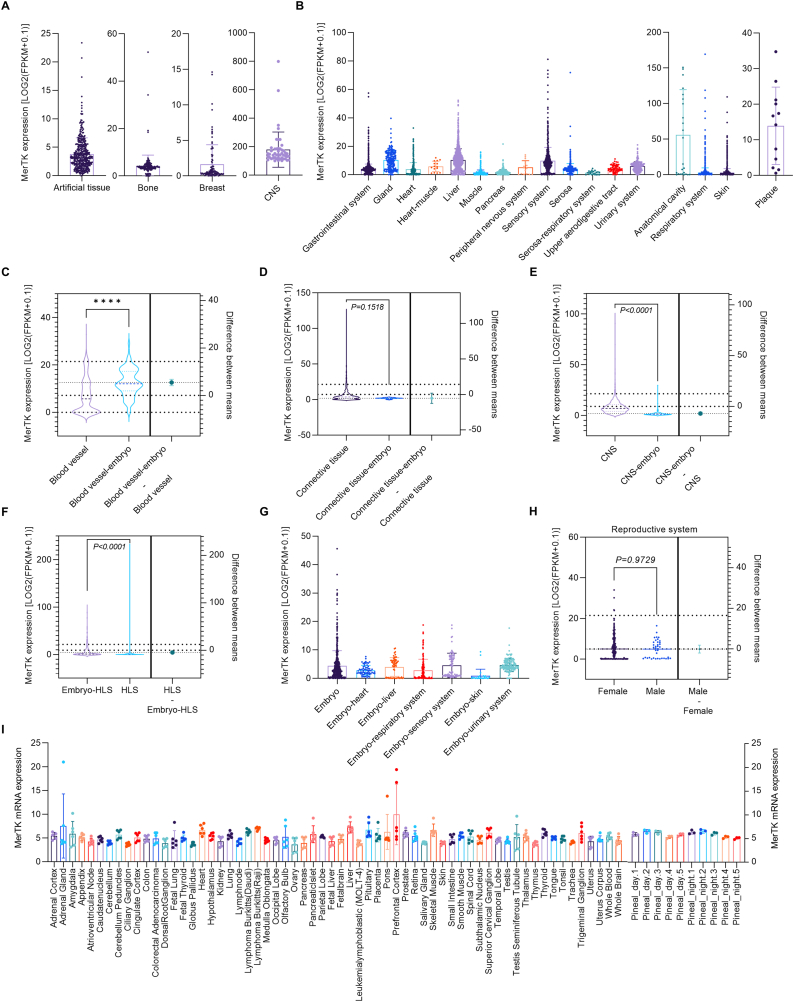
**MerTK expression in human tissues.** RNA-seq or scRNA-seq for MerTK expression in (**A**–**B**) human tissues sorted alphabetically, (**C**–**G**) human tissues from adult and embryo, and (**H**) human reproductive system from female and male. (**I**) MerTK mRNA expression in human tissues from BioGPS. Original data of RNA-seq or scRNA-seq for MerTK expression, quantified by Log2 (FPKM + 0.1), were downloaded from QIAGEN OmicSoft Land Explorer. The data were analyzed with GraphPad Prism 9.4.1 and shown as the mean ± SD. An unpaired *t*-test was used to compare the mean between two groups. P < 0.05 was considered statistically significant.

MerTK is known to play a fundamentally protective role in cardiovascular disease (e.g., atherosclerosis, myocardial infarction, and vascular aging), brain disorders, cancers, other pathologies by promoting efferocytosis, inflammation resolution, and vascular remodeling [[1], [2], [3],^5^,^10^,^11^,[13], [14], [15],^21^]. Therefore, we performed big data analytics for MerTK expression in the human tissues involved in these diseases. The data revealed that MerTK is highly expressed in a variety of human tissues sorted alphabetically (Fig. 2A–B). Extensive expression of MerTK also is found in embryo tissues, including blood vessel (Fig. 2C), connective tissue (Fig. 2D), CNS (Fig. 2E), hematopoietic and lymphoid system (HLS) (Fig. 2F), and other embryo tissues including heart, liver, respiratory system, sensory system, skin, and urinary system (Fig. 2G). Interestingly, MerTK expression in embryo blood vessel is 75.8 % higher (p < 0.0001) than in normal adult blood vessel (Fig. 2C). We also found extraordinarily high expression of MerTK in CNS and MerTK expression is 345.44 % higher (p < 0.0001) in normal adult CNS than embryo CNS (Fig. 2E). There is no significant difference in MerTK expression in connective tissue between normal and embryo (Fig. 2D). However, the difference is significant between normal and embryo in HLS (Fig. 2F). In the reproductive system, there is no difference in MerTK expression between male and female (Fig. 2H). Consistent with the RNA-seq data, the BioGPS data showed the ubiquitous expression of MerTK transcript in human tissues (Fig. 2I), indicating the potential impact of MerTK on the function of many different human tissues and related diseases that could be influenced by MerTK dysregulation.

### 3.3 MerTK and human tissue-specific diseases

**Fig. 3:**
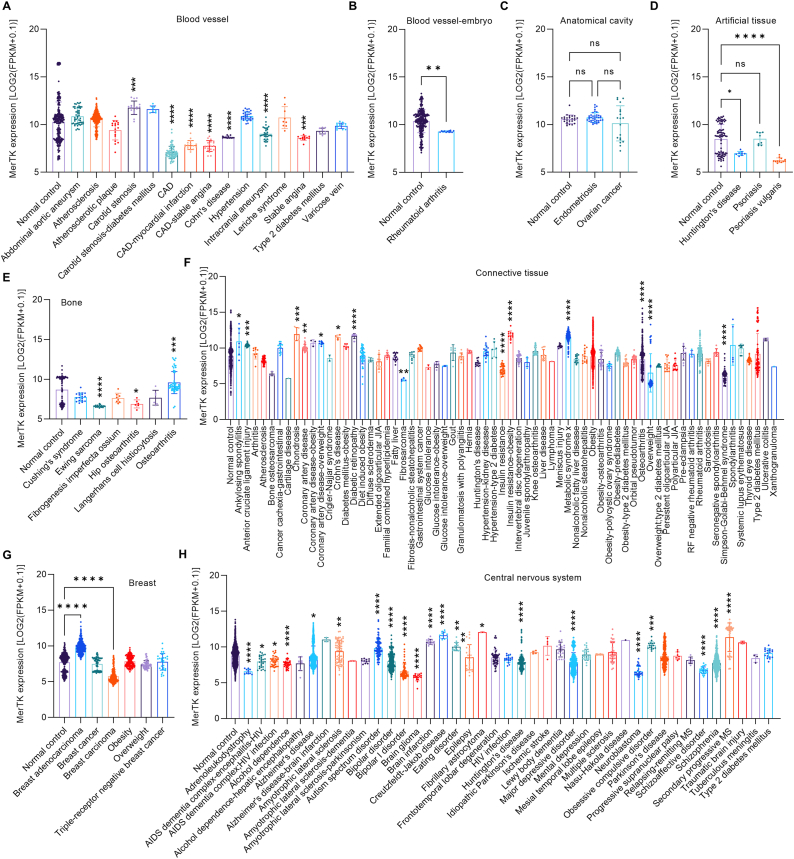
**MerTK expression in human diseases specifically in related tissues**, including (**A**–**B**) blood vessel and embryo blood vessel, (**C**) anatomical cavity, (**D**) Artificial tissue, (**E**) Bone, (**F**) Connective tissue, (**G**) Breast, and (**H**) Central nervous system. MerTK expression was based on RNA-seq or scRNA-seq and was quantified by Log2 (FPKM + 0.1). Original data of RNA-seq or scRNA-seq for MerTK expression, quantified by Log2 (FPKM + 0.1), were downloaded from QIAGEN OmicSoft Land Explorer. The data were analyzed with GraphPad Prism 9.4.1 and shown as the mean ± SD. Dunnett's one-way ANOVA was used for multiple comparisons between disease types and normal control. P < 0.05 was considered statistically significant.

Next, we investigated MerTK expression in a wide range of human diseases. In blood vessel diseases compared with normal control, MerTK expression is 14.2 % (p = 0.0002) higher in carotid stenosis but significantly lower in coronary artery disease (CAD), CAD coupled to myocardial infarction or stable angina, Cohn's disease, intracranial aneurysm, and stable angina (Fig. 3A). In embryo blood vessels, MerTK expression is 11.8 % (p = 0.0022) lower in rheumatoid arthritis than in the control group (Fig. 3B). There is no difference in anatomical cavity with endometriosis and ovarian cancer compared with normal control (Fig. 3C). Artificial tissues, also known as biomaterials, are synthetically created tissues that can be implanted or integrated into the patients to replace the damaged or non-functional tissues. In artificial tissues, we found that MerTK expression is 21.4 % (p = 0.0124) and 35.2 % (p < 0.0001) lower in Huntington's disease and psoriasis vulgaris, respectively (Fig. 3D). MerTK is highly expressed in bone marrow–derived macrophages and determines efferocytosis [1], inferring a potential role for MerTK in bone diseases. Expression is 9.2 % (p = 0.0008) higher in osteoarthritis but 31.5 % lower (p < 0.0001) in Ewing sarcoma and 25.9 % lower (p = 0.0155) in hip osteoarthritis (Fig. 3E). Connective tissue refers to several bodily tissues that support or connect other tissues [22] and our big data analytics show a significant difference of MerTK expression between many types of control vs diseased connective tissues (Fig. 3F). In breast disease, MerTK is expressed significantly higher in breast adenocarcinoma but lower in breast carcinoma (Fig. 3G). The CNS is the neuro-hub responsible for receiving, integrating, and responding to sensory information. Brain tumors, Parkinson's disease, Alzheimer's disease, and stroke are common CNS disorders in human. In this regard, MerTK expression is broadly abnormal in a large variety of CNS disorders including adrenoleukodystrophy, acquired immunodeficiency syndrome (AIDS), alcohol dependence, Alzheimer's disease, Huntington's disease, major depressive disorder, neuroblastoma, obsessive compulsive disorder, schizoaffective disorder, and schizophrenia (Fig. 3H).

**Fig. 4:**
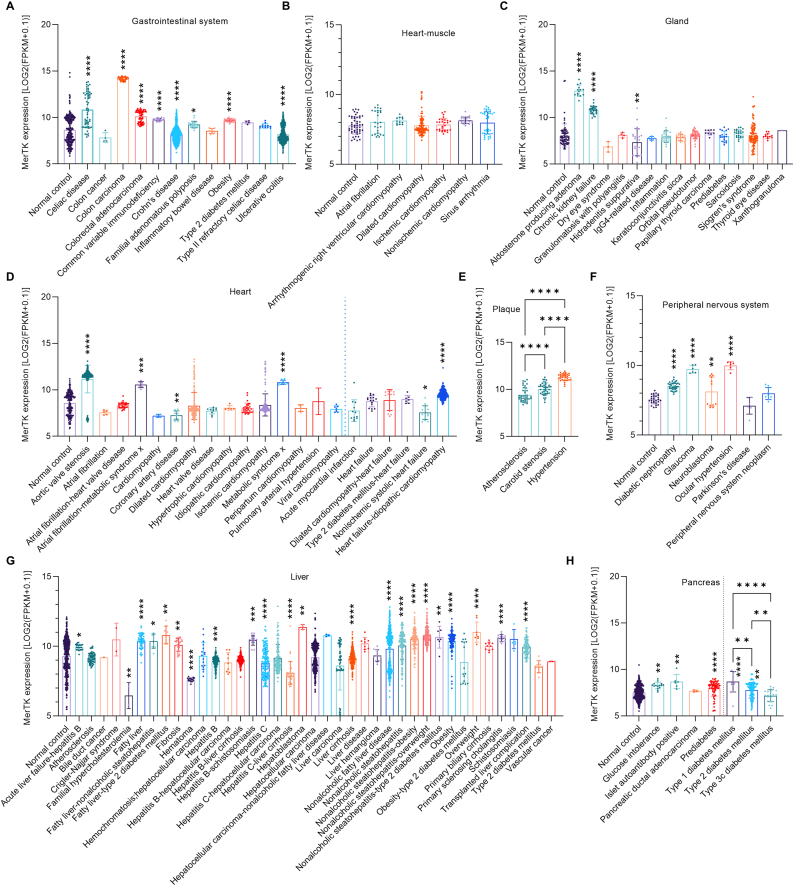
**MerTK expression in human diseases specifically in related tissues**, including (**A**) Gastrointestinal system, (**B**) Heart muscle, (**C**) Gland, (**D**) Heart, (**E**) Plaque, (**F**) Peripheral nervous system, (**G**) Liver, and (**H**) Pancreas. MerTK expression was based on RNA-seq or scRNA-seq and was quantified by Log2 (FPKM + 0.1). Original data of RNA-seq or scRNA-seq for MerTK expression, quantified by Log2 (FPKM + 0.1), were downloaded from QIAGEN OmicSoft Land Explorer. The data were analyzed with GraphPad Prism 9.4.1 and shown as the mean ± SD. Dunnett's one-way ANOVA was used for multiple comparisons between disease types and normal control. P < 0.05 was considered statistically significant.

The gastrointestinal system comprises the gastrointestinal tract and accessory organs that are responsible for digestive functions [23]. We found that MerTK expression is moderately to highly increased in the gastrointestinal system during related human diseases such as celiac disease, colon carcinoma, colorectal adenocarcinoma, common variable immunodeficiency, and obesity (Fig. 4A). However, MerTK is lowly expressed in the gastrointestinal system during Crohn's disease, familial adenomatous polyposis, and ulcerative colitis (Fig. 4A). In heart disorders implicated diseases including atrial fibrillation, cardiomyopathy disorders, and sinus arrhythmia, there is no difference in MerTK expression between normal and disease groups (Fig. 4B). A gland is a group of cells that secretes or excretes chemical substances (e.g., hormones) and releases them into the bloodstream (endocrine gland) or into cavities. In gland diseases, we found that, compared with normal control, MerTK expression is 37.4 % higher (p < 0.0001) in aldosterone producing adenoma and 25.4 % higher (p < 0.0001) chronic kidney failure, while is 10.1 % lower (p = 0.0049) in hidradenitis suppurativa (Fig. 4C). In heart disease, MerTK expression is significantly higher in aortic valve stenosis, atrial fibrillation-metabolic syndrome, metabolic syndrome, and heart failure-idiopathic cardiomyopathy (Fig. 4D). In contrast, MerTK is lowly expressed in coronary artery disease, acute myocardial infarction, and nonischemic systolic heart failure (Fig. 4D). In plaque, compared with atherosclerosis alone group, MerTK significantly highly expressed in atherosclerosis accompanied with carotid stenosis and hypertension (Fig. 4E). The peripheral nervous system consists of the nerves and ganglia outside of the brain and spinal cord. MerTK expression in the peripheral nervous system is significantly increased in diabetic nephropathy, glaucoma, and ocular hypertension, whereas expression is 6.8 % lower (p = 0.0071) than control in neuroblastoma (Fig. 4F). There are many types of liver disease, including alcohol-related liver disease, hepatitis A/B/C, fatty liver disease, cirrhosis, and liver cancer. We found that MerTK is extensively expressed in a variety of liver diseases shown in Fig. 4G. MerTK is expressed significantly less in familial hypercholesterolemia, hematoma, and hepatitis B/C (Fig. 4G), indicating MerTK restoration may be a potential therapeutic treatment for these liver diseases. However, MerTK is upregulated in many other liver diseases (Fig. 4G). The pancreas is a common site of disease in the digestive system and some common afflictions include cystic fibrosis, pancreatic cancer, and pancreatitis. Fig. 4H shows that expression of MerTK is significantly increased in glucose intolerance, islet autoantibody positive, and prediabetes. Interestingly, MerTK has different expression patterns in the pancreas in different types of diabetes mellitus. For example, MerTK expression is 11.7 % higher (p = 0.0021) in type 1 diabetes mellitus but is 17.5 % lower (p < 0.0001) in type 2 diabetes and 8.4 % lower (p = 0.0088) in type 3c diabetes mellitus (Fig. 4H).

**Fig. 5:**
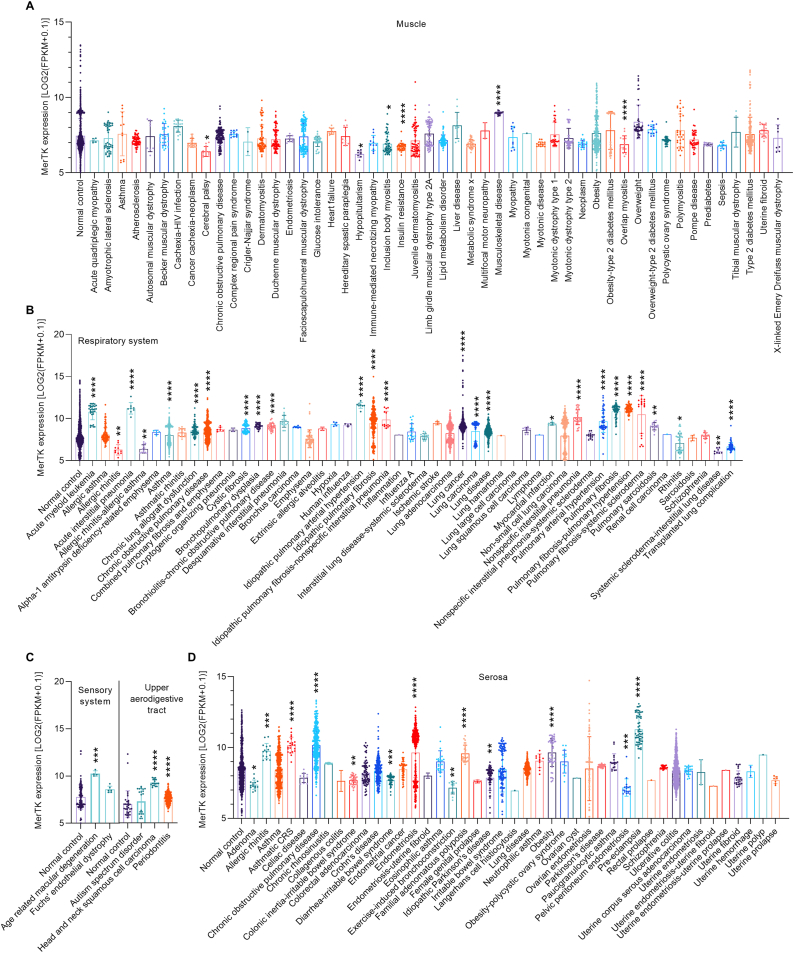
**MerTK expression in human diseases specifically in related tissues**, including (**A**) Muscle, (**B**) Respiratory system, (**C**) Sensory system and upper aerodigestive tract, and (**D**) Serosa. Original data of RNA-seq or scRNA-seq for MerTK expression, quantified by Log2 (FPKM + 0.1), were downloaded from QIAGEN OmicSoft Land Explorer. The data were analyzed with GraphPad Prism 9.4.1 and shown as the mean ± SD. Dunnett's one-way ANOVA was used for multiple comparisons between disease types and normal control. P < 0.05 was considered statistically significant.

**Fig. 6:**
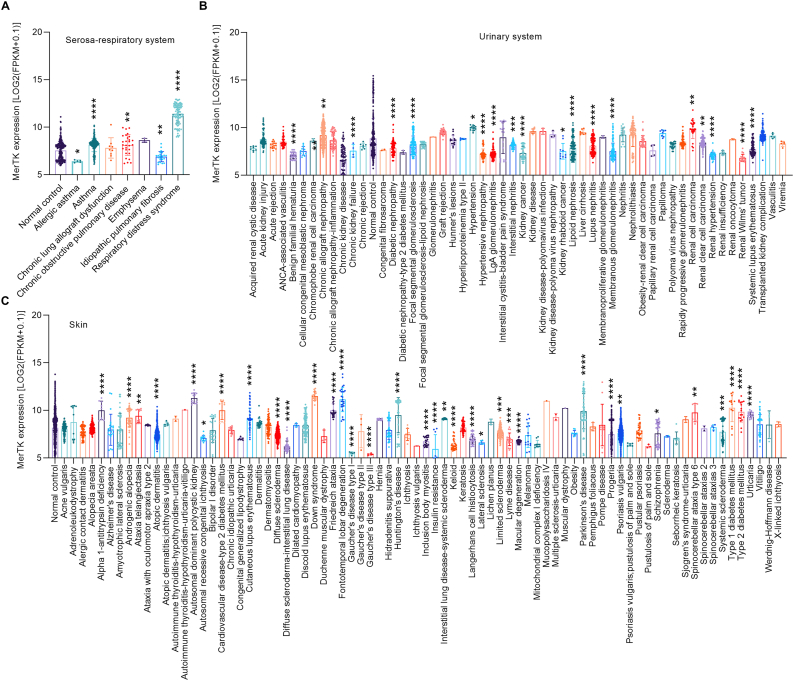
**MerTK expression in human diseases specifically in related tissues**, including (**A**) Serosa-respiratory system, (**B**) Urinary system, and (**C**) Skin. Original data of RNA-seq or scRNA-seq for MerTK expression, quantified by Log2 (FPKM + 0.1), were downloaded from QIAGEN OmicSoft Land Explorer. The data were analyzed with GraphPad Prism 9.4.1 and shown as the mean ± SD. Dunnett's one-way ANOVA was used for multiple comparisons between disease types and normal control. P < 0.05 was considered statistically significant.

Muscle disease can refer to diseases of the cardiac, skeletal or smooth muscle cells that often manifest as loss of muscle function with site-specific symptoms [24]. MerTK expression in muscle only is altered in certain diseases including cerebral palsy, inclusion body myositis, insulin resistance, musculoskeletal disease, overlap myositis, and obesity (Fig. 5A). Respiratory illness also afflicts many people worldwide and common diseases include asthma and chronic obstructive pulmonary disease (COPD) [25,26]. We found that MerTK is significantly upregulated in asthma, COPD, pulmonary arterial hypertension, pulmonary fibrosis, and other respiratory system related diseases (as indicated), but is expressed significantly less in allergic rhinitis, allergic rhinitis allergic asthma, rhinitis, systemic scleroderma-interstitial lung disease, and transplanted lung complication (Fig. 5B). The sensory system is a nervous system component consisting of sensory receptors that are responsible for processing sensory stimuli to elicit appropriate physiological responses [27,28]. There are very limited RNA-seq data related to MerTK biology and human disease related to the sensory system and upper aerodigestive tract. We summarized existing RNA-seq data and showed that, compared with normal control, MerTK is highly expressed (24.2 %, p = 0.0003) in age related macular degeneration and highly expressed in head and neck squamous cell carcinoma (24.5 %, p < 0.0001), and periodontitis (8.4 %, p < 0.0001) (Fig. 5C). Serosa consists of an epithelial layer and a connective tissue layer, which allows lubricated sliding movements between opposing surfaces by secreting serous fluid [29]. MerTK has been shown to play a role in human serosa disorders. Our big data analytics of RNA-seq showed that, compared with normal control, MerTK expression is significantly higher in many serosa disorders (Fig. 5D). Serosa is also a key component of the human respiratory system [30] and MerTK expression in serosa is much higher in the respiratory diseases of asthma, COPD, and respiratory distress syndrome, and lower in disease conditions such as allergic asthma and idiopathic pulmonary fibrosis (Fig. 6A). Common diseases of the urinary system include nephrosis (noninflammatory kidney disease), nephrolithiasis (kidney stones), urethritis (urethral inflammation), nocturia, and enuresis [31]. We found abnormal expression of MerTK in many urinary system-related diseases (Fig. 6B). For example, MerTK is more highly expressed in chronic allograft nephropathy, focal segmental glomerulosclerosis, and renal cell carcinoma, while expressed less in benign familial hematuria, chronic kidney failure, and renal Wilms' tumor (Fig. 6B). Finally, we analyzed MerTK expression in skin, the largest organ of the body, and related human diseases [32]. Common skin disorders include acne, eczema, psoriasis, Raynaud's phenomenon, rosacea, skin cancer, and vitiligo [32]. MerTK expression also is modulated during skin disorders (Fig. 6C). Interestingly, MerTK expression is lower in Gaucher's disease type I (67.3 %, p < 0.0001) and III (65.9 %, p < 0.0001), but not changed in Gaucher's disease type II (Fig. 6C). On the contrary, MerTK expression is significantly higher in skin of humans with autosomal dominant polycystic kidney, Down syndrome, frontotemporal lobar degeneration, type ½ diabetes mellitus, and urticaria (Fig. 6C).

### 3.4 Sex differences in MerTK expression in human

**Fig. 7:**
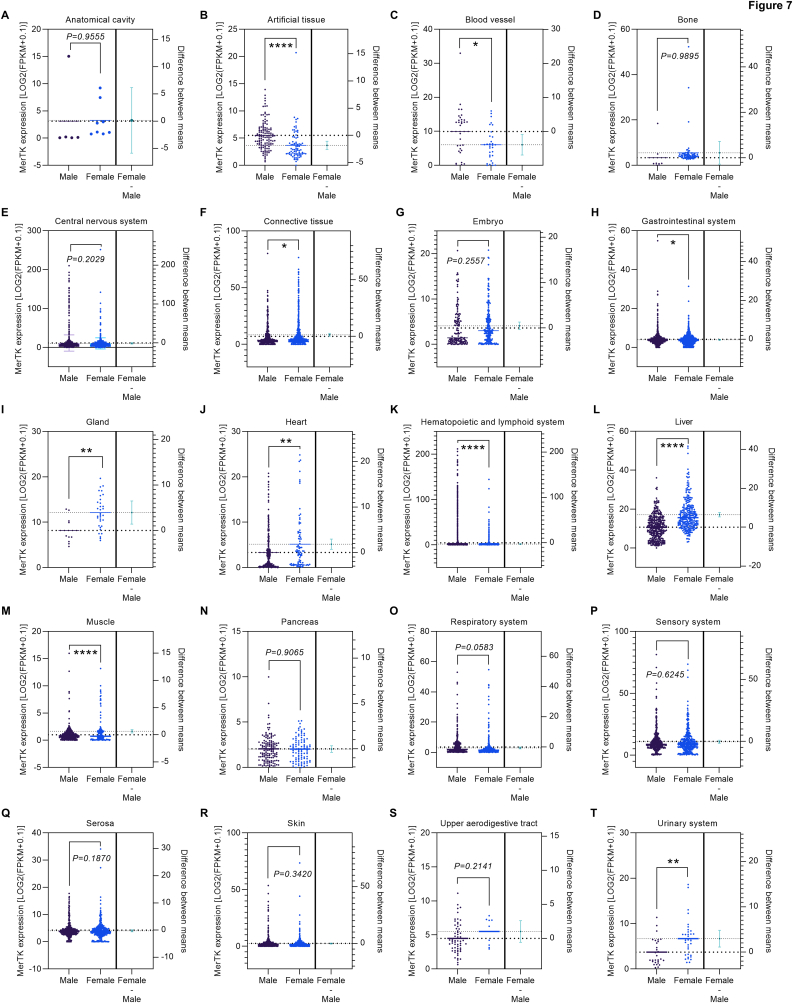
**MerTK expression in human tissues from male and female sorted alphabetically,** including (**A**) Anatomical cavity, (**B**) Artificial tissue, (C) Blood vessel, (**D**) Bone, (**E**) Central nervous system, (**F**) Connective tissue, (**G**) Embryo, (**H**) Gastrointestinal system, (**I**) Gland, (**J**) Heart, (**K**) Hematopoietic and lymphoid system, (**L**) Liver, (**M**) Muscle, (**N**) Pancreas, (**O**) Respiratory system, (**P**) Sensory system, (**Q**) Serosa, (**R**) Skin, (**S**) Upper aerodigestive tract, and (**T**) Urinary system. Original data of RNA-seq or scRNA-seq for MerTK expression, quantified by Log2 (FPKM + 0.1), were downloaded from QIAGEN OmicSoft Land Explorer. The data were analyzed with GraphPad Prism 9.4.1 and shown as the mean ± SD. An unpaired *t*-test was used to compare the mean between male and female. P < 0.05 was considered statistically significant.

**Fig. 8:**
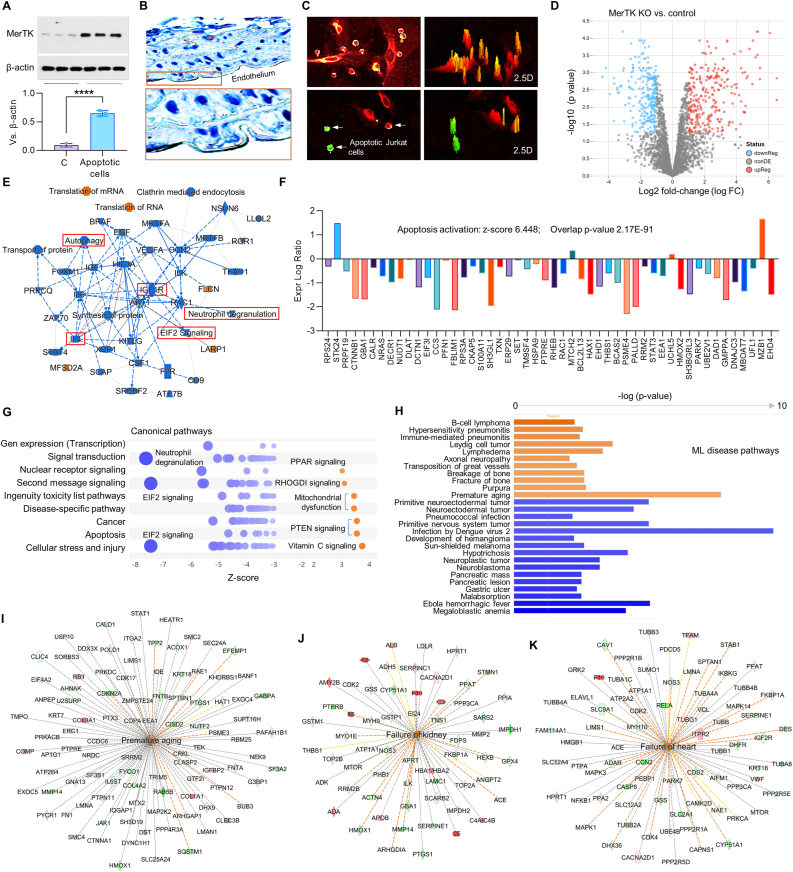
**Proteomics in HAECs with MerTK gene knockout or control.** (**A**) MerTK expression in HAECs incubated with apoptotic Jurkat cells for 1 h. (**B**) Immunochemical staining for MerTK expression in the aortic arch from WT mice. (**C**) Efferocytosis of apoptotic Jurkat cells by HAECs after 1 h of co-incubation. P < Apoptotic Jurkat cells were labeled with green PKH67 (Sigma) and HAECs were labeled with red PKH26 (Sigma). Green cells are apoptotic Jurkat cells that were not engulfed by HAECs. Green/red small round cells are apoptotic Jurkat cells that were engulfed by HAECs. Large red cells are HAECs. (D) Volcano plot illustration in MerTK KO vs. control. Relative protein abundance (log2) plotted against significance level (-log10 P-value), showing significantly (p < 0.05) downregulated (blue), upregulated (red) or non-differentially expressed proteins (grey). (E) Graphic summarization for pathways in MerTK KO vs. control. (F) MerTK KO activates apoptosis signaling. (G) Canonical pathway analysis in MerTK KO vs. control. Color depends on z-score. Blue signifies negative value; orange signifies positive value; and grey signifies no activity pattern. Size is proportional to the number of genes that overlap the pathway. (H) Machine learning analysis for activated or inhibited disease pathways. (I–K) IPA prediction shows that MerTK KO activates premature aging, kidney failure and heart failure. Proteomics data were analyzed by IPA. Data were analyzed with GraphPad Prism 9.4.1 and shown as the mean ± SD (n = 3–5). P < 0.05 was considered statistically significant. (For interpretation of the references to color in this figure legend, the reader is referred to the Web version of this article.)

Although we observed that MerTK expression in the reproductive systems of male and female is not different (Fig. 2H), we posited that MerTK expression in other tissues may differ significantly between male and female. Therefore, we investigated this possibility using RNA-seq or scRNA-seq analysis (Fig. 7). We found that there is no difference between male and female for MerTK expression in tissues of anatomical cavity (Fig. 7A), bone (Fig. 7D), CNS (Fig. 7E), embryo (Fig. 7G), pancreas (Fig. 7N), respiratory system (Fig. 8C), sensory system (Fig. 7P), serosa (Fig. 7Q), skin (Fig. 7R), and upper aerodigestive tract (Fig. 7S). However, MerTK expression was 33.2 % higher (p < 0.0001) in male than female in artificial tissues (biomaterials, Figs. 7B), 39.2 % higher (p = 0.0122) in blood vessel (Figs. 7C), 5.9 % higher (p = 0.0323) in the gastrointestinal system (Figs. 7H), 59.3 % higher (p < 0.0001) in the hematopoietic and lymphoid system (Figs. 7K), and 62.5 % higher (p < 0.0001) in muscle (Fig. 7M). Thirdly, we found that MerTK expression was 16.4 % lower (p = 0.022) in male than female in tissues of connective tissue (Figs. 7F), 32.5 % lower (p = 0.0033) in gland (Figs. 7I), 35.6 % lower (p = 0.0015) in heart (Figs. 7J), 37.8 % lower (p < 0.0001) in liver (Figs. 7L), and 44.5 % lower (p = 0.002) in the urinary system (Fig. 7T). These interesting and novel data suggest that sex difference should be taken into consideration when defining individual risk for MerTK targeted therapy.

### 3.5 Proteomics analysis for MerTK functions

Human Jurkat cells, an acute T lymphocytes cell line with ∼10. 72 μm in diameter, are widely used for MerTK-mediated efferocytosis assays in vitro [2,5]. To validate the functions of MerTK in human diseases, we performed proteomics in primary human aortic ECs (HAECs) with or without incubation of apoptotic Jurkat cells. First, we provided evidence that MerTK is markedly induced by apoptotic Jurkat cells in HAECs (Fig. 8A) and highly expressed in endothelium from the aortic arch (Fig. 8B). Our immunostaining results showed that HAECs have high ability to perform efferocytosis for the clearance of apoptotic Jurkat cells (Fig. 8C). Second, we investigated MerTK functions at protein level based on proteomics in HAECs that were transfected with MerTK CRISPR/Cas9 knockout (KO) Plasmid or control plasmid. Fig. 8D shows volcano plot illustration with a clear separation in protein profiles between MerTK KO and control group. A total of 673 proteins including 356 upregulated proteins and 317 downregulated proteins in the MerTK KO group were identified. Fig. 8E shows the graphical summary for the signaling pathways in MerTK KO vs. control. Compared with control, MerTK KO inhibits a series of signaling pathways, such as autophagy (a self-degradative process), IGF1R (insulin-like growth factor 1), IL-4 (anti-inflammatory cytokine), EIF2 (eukaryotic Initiation Factor 2), and neutrophil degranulation. While MerTK KO activates translation of mRNA and RNA. MerTK KO induces apoptosis activity with an activation z-score of 6.448 that are detailly shown in Fig. 8F. Our canonical pathway analysis shows that MerTK KO activates PPAR (peroxisome proliferator-activated receptors) signaling, RHOGDI (Rho GDP-dissociation inhibitor) signaling, mitochondrial dysfunction, PTEN (phosphatase and tensin homolog) signaling, and vitamin C signaling; while MerTK KO inhibits neutrophil degranulation, EIF2 signaling, and other signaling (Fig. 8G). Machine learning (ML) disease pathways analysis shows the consequence of MerTK KO in human diseases, such as activated diseases (e.g., B-cell lymphoma, pneumonitis and aging) or inhibited diseases (e.g., infection by dengue virus 2, hypotrichosis and megaloblastic anemia) (Fig. 8H). Finally, IPA prediction shows that MerTK KO promotes premature aging, kidney failure and heart failure, which is consistent with our big data analytics (Fig. 8I–K).

### 3.6 IPA for ADAM17 based on scRNA-seq

**Fig. 9:**
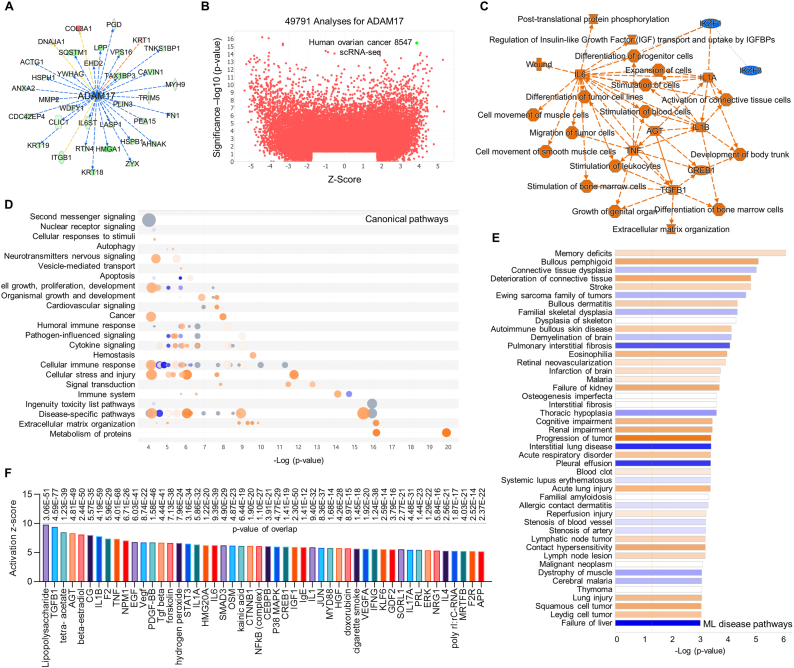
**scRNA-seq for ADAM17 in human ovarian cancer 8547 based on IPA database.** (**A**) Effect of MerTK KO on ADAM17 signaling pathways in HAECs based on IPA prediction in proteomics. (**B**) Activity plot for ADAM17 including 49791 analyses based on the omics database from QIAGEN IPA and QIAGEN OmicSoft Land Explorer. (**C**) Summary signaling pathways. Orange and blue indicate activation and inhibition, respectively. Grey dashed lines depict no prediction. (**D**) Canonical pathways based on a -log(p-value) greater than 4.0. Color depends on z-score. Blue signifies negative value; orange signifies positive value; and grey signifies no activity pattern. Size is proportional to the number of genes that overlap the pathway. (E) Machine learning analysis for activated or inhibited disease pathways based on a -log(p-value) greater than 3.0. (For interpretation of the references to color in this figure legend, the reader is referred to the Web version of this article.)

ADAM17 (a disintegrin and metalloprotease 17) is responsible for cleavage of MerTK, resulting in defective efferocytosis that contributes to accumulation of apoptotic cells and tissue injury in cardiovascular diseases including atherosclerosis [3]. Interestingly, our proteomics showed that MerTK KO inhibits ADAM17, indicating a negative regulation between MerTK and ADAM17 (Fig. 9A). To further investigate the role of ADAM17 in human disease, the omics database from QIAGEN IPA and QIAGEN OmicSoft Land Explorer were applied. Based on the activation z-score, 49791 analyses for ADAM17 were identified, providing the rational of human ovarian cancer 8547 as our analysis target because it has the highest combination of significance -log10 (p-value) and activation z-score of ADAM17 (Fig. 9B). The summary of signaling pathway shows that ADAM17 activation is associated with many signaling pathways, such as inflammation response (e.g. IL-6, IL-1β, TNF, and TGF-β1), migration of tumor cells, tissue wound, and extracellular matrix organization (Fig. 9C). The canonical pathways show that ADAM17 activation is accompanied with cancer, cellular stress and injury, extracellular matrix organization, metabolism of proteins and other signaling pathways (Fig. 9D). The ML disease pathways analysis shows that ADAM17 activation promotes memory deficits, bullous pemphigoid, deterioration of connective tissue, stroke and human diseases (Fig. 9E). Fig. 9F shows the top 50 upregulated signaling followed with ADAM17 activation, such as lipopolysaccharide (LPS), TGF-β1, IL-1β, NF-κB, p38 MAPK and IFN-γ, all are involved in cardiovascular dysfunction, brain disorders and cancer development.

## 4 Discussion

RNA-seq or scRNA-seq is a powerful sequencing technique used to reveal the presence and quantity of RNA in a biological sample for transcriptomic analysis. To our knowledge, the present study provides the first analysis of MerTK gene expression in human diseases based on RNA-seq or scRNA-seq. We detail the expression of the MerTK receptor in human cells and tissues from both male and female and in a wide variety of human diseases; the dynamic balance of MerTK expression may depend on cell and tissue types, disease states, and sex difference. Our big data analytics show that MerTK is extensively expressed in cardiovascular diseases, brain disorders and cancer, which account for the major causes of death worldwide. In the cardiovascular system, MerTK is highly expressed in cardiovascular cells (e.g., ECs, SMCs, and macrophages) and cardiovascular tissues (e.g., plaque, blood vessel and heart) [5,33], indicating the potential role of MerTK in cardiovascular diseases. An abnormally low expression of MerTK is found in myocardial infarction and stable angina, and intracranial aneurysm. As the main receptor for efferocytosis, lower MerTK expression would be expected to cause impaired efferocytosis, accumulation of apoptotic and necrotic cells, and inflammation, which are processes associated with atherosclerosis, myocardial infarction, and aneurysm rupture [11,33]. In brain, efferocytosis is mainly performed by brain-resident microglia and/or infiltrating macrophages, which contribute to the clearance of apoptotic and necrotic cells [34]. In addition to microglia, neuronal progenitor cells (e.g., astrocytes) and vascular cells (e.g., ECs and SMCs) also participate in the removal of apoptotic and necrotic cells [33,34]. Similar to the cardiovascular system, our big data analytics show that MerTK expression is significantly lower in several common brain disorders including brain glioma, neuroblastoma, and schizoaffective disorder. Different than normal cells, tumor cells may induce a tumor-tolerant environment with the aid of the anti-inflammatory effect of efferocytosis. For example, in breast cancer, efficient efferocytosis resulting in brisk apoptotic cell clearance may have deleterious consequences, such as facilitating tumor growth and increasing the immunosuppressive tumor micro-environment [35]. In this regard, it is interesting that MerTK is highly expressed in breast adenocarcinoma but lowly expressed in common breast carcinoma, implying that the beneficial versus detrimental functions of MerTK mediated by efferocytosis may be cancer subtype-specific in impact.

An interesting finding related to MerTK is its expression in immune cells. The most common immune cells include T cells, B cells, natural killer cells (NK) cells, neutrophils, macrophages, and other monocytes. To date, there are very limited studies focusing on MerTK function in immune cells. Cabezo'n et al. found that MerTK in dendritic cells is a potent suppressor of human T cell activation [36]. Peeters et al. reported that MerTK serves as a late costimulatory signal for CD8^+^ T cells [37]. Findings by Lindsay et al. indicate that MerTK in monocytes from the pancreatic islets regulates T cell activation in type 1 diabetes and melanoma, leading to decreased T cell scanning sensitivity for cognate antigen [38]. Giroud et al. showed that MerTK is expressed in activated T and B cells, revealing that MerTK may potentially regulate dendritic cell activation [39]. Giroud et al. also showed that, compared with Tyro3 and Axl, MerTK is the most prominent TAM receptor expressed in immunosuppressive myeloid cells [39]. Consistent with these findings, our data analytics confirmed that MerTK is highly expressed in activated plasma B cells responding to tumor environment but is lowly expressed in alpha-beta T cells, CD4^+^ central and effector memory T cells, and CD4^+^CD8^+^ T cells. Compared with T cells, it seems that MerTK is higher expressed in several types of B cells, including germinal center B cells and common B cells. Cancer cells are known to highly express ‘‘don't eat me’’ signals such as CD47 that help them escape efferocytosis, and as discussed earlier, MerTK may facilitate tumor growth in some cancerous conditions [40]. In these circumstances, MerTK gene therapy may enable immune cells to improve cell efferocytosis and enhance therapeutically exploit cancer immunotherapy to enhance immune cell clearance of dying cancer cells.

**Clinical Perspectives. *Competency in medical knowledge.*** MerTK inhibitor is a novel anti-cancer target due to its aberrant expression in numerous human malignancies. However, MerTK may undergo cleavage resulting in defective efferocytosis in cardiovascular diseases, brain disorders, and other diseases, making MerTK restoration a promising therapeutic strategy to treat these disease conditions. Interestingly, we also provide the first evidence that there are tissue-specific sex differences in MerTK expression. These differences should be taken into consideration when defining individual risk for different disease events and when contemplating MerTK inhibition/restoration treatments. However, it's not clear whether the changes in the expression of MerTK were controlled by disease type or disease severity. More clinical studies need to be performed in patients with identical disease type and disease severity. ***Translational outlook.*** MerTK may represent a promising genetic target for drug design with careful consideration of its disease type- and sex-dependent effects. Our big data analytics provide further rationale for continued development of MerTK-targeted therapeutic drugs for clinical trials, which are needed to confirm the safety and efficacy of these drugs.

**Study strengths, limitations, and conclusions.** In these big data analytics, we provide the first summary of MerTK transcript expression in a wide range of human cells, tissues, and disease states as well as initial data in males and females. Increasing evidence indicates that anti-MerTK medications may represent an emerging strategy to treat chronic inflammatory and autoimmune disorders. The central principle is to enhance cell efferocytosis, driving clearance of apoptotic bodies, promoting inflammation resolution, and suppressing anti-tumor immunity. It is worth noting that in myocardial infarction, a leading cause of death worldwide, MerTK-mediated efficient clearance of dying cardiomyocytes is beneficial for restoration of cardiac function [11]. However, our findings of MerTK expression in this report need to be cross-validated by q-PCR, Western blotting, and immunostaining and definition of MerTK cellular and molecular mechanisms investigated using CRISPR/Cas9 KO plasmid, CRISPR activation plasmid, or conditional knockout animals that are commercially available or custom designed. Our big data analytics for MerTK are intended to provide novel evidence and reference opinions for the development and application of MerTK-targeted therapeutics to treat human diseases characterized by excessive accumulation of apoptotic cells, impaired efferocytosis, and subsequent defective inflammation resolution.

## CRediT authorship contribution statement

**Shijie Liu:** Conceptualization, Data curation, Formal analysis, Writing – original draft. **Jinzi Wu:** Methodology, Data curation. **Daixuan Yang:** Writing – review & editing. **Jianliang Xu:** Writing – review & editing. **Hang Shi:** Writing – review & editing. **Bingzhong Xue:** Writing – review & editing. **Zufeng Ding:** Conceptualization, Data curation, Funding acquisition, Methodology, Project administration, Resources, Software, Supervision, Validation, Writing – original draft, Writing – review & editing.

## Declaration of competing interest

The authors have declared that no competing interest exists.

## Data Availability

Data will be made available on request.

## References

[1] Myers K.V., Amend S.R., Pienta K.J. (2019). Targeting Tyro3, Axl and MerTK (TAM receptors): implications for macrophages in the tumor microenvironment. Mol. Cancer.

[2] Cai B., Thorp E.B., Doran A.C., Sansbury B.E., Daemen M.J., Dorweiler B., Spite M., Fredman G., Tabas I. (2017). MerTK receptor cleavage promotes plaque necrosis and defective resolution in atherosclerosis. J. Clin. Invest..

[3] Thorp E., Vaisar T., Subramanian M., Mautner L., Blobel C., Tabas I. (2011). Shedding of the Mer tyrosine kinase receptor is mediated by ADAM17 protein through a pathway involving reactive oxygen species, protein kinase Cδ, and p38 mitogen-activated protein kinase (MAPK). J. Biol. Chem..

[4] Seitz H.M., Camenisch T.D., Lemke G., Earp H.S., Matsushima G.K. (2007). Macrophages and dendritic cells use different Axl/Mertk/Tyro3 receptors in clearance of apoptotic cells. J. Immunol..

[5] Liu S., Wu J., Stolarz A. (2023). PCSK9 attenuates efferocytosis in endothelial cells and promotes vascular aging. Theranostics.

[6] Lee Y.J., Park M., Kim H.Y. (2023). Circulating small extracellular vesicles promote proliferation and migration of vascular smooth muscle cells via AXL and MerTK activation. Acta Pharmacol. Sin..

[7] Huelse J.M., Fridlyand D.M., Earp S., DeRyckere D., Graham D.K. (2020). MERTK in cancer therapy: targeting the receptor tyrosine kinase in tumor cells and the immune system. Pharmacol. Ther..

[8] Cook R.S., Jacobsen K.M., Wofford A.M. (2013). MerTK inhibition in tumor leukocytes decreases tumor growth and metastasis. J. Clin. Invest..

[9] Giroud P., Renaudineau S., Gudefin L. (2020). Expression of TAM-R in human immune cells and unique regulatory function of MerTK in IL-10 production by tolerogenic DC. Front. Immunol..

[10] Pierce A.M., Keating A.K. (2014). TAM receptor tyrosine kinases: expression, disease and oncogenesis in the central nervous system. Brain Res..

[11] DeBerge M., Yeap X.Y., Dehn S. (2017). MerTK cleavage on resident cardiac macrophages compromises repair after myocardial ischemia reperfusion injury. Circ. Res..

[12] Al-Zaeed N., Budai Z., Szondy Z., Sarang Z. (2021). TAM kinase signaling is indispensable for proper skeletal muscle regeneration in mice. Cell Death Dis..

[13] Cummings C.T., DeRyckere D., Earp H.S., Graham D.K. (2013). Molecular pathways: MERTK signaling in cancer. Clin. Cancer Res..

[14] Konishi H., Okamoto T., Hara Y. (2020). Astrocytic phagocytosis is a compensatory mechanism for microglial dysfunction. EMBO J..

[15] Miner J.J., Daniels B.P., Shrestha B. (2015). The TAM receptor Mertk protects against neuroinvasive viral infection by maintaining blood-brain barrier integrity. Nat Med.

[16] Wu H., Zheng J., Xu S. (2021). Mer regulates microglial/macrophage M1/M2 polarization and alleviates neuroinflammation following traumatic brain injury. J. Neuroinflammation.

[17] Krämer A., Green J., Pollard J., Tugendreich S. (2014). Causal analysis approaches in Ingenuity pathway analysis. Bioinformatics.

[18] Yunna C., Mengru H., Lei W., Weidong C. (2020). Macrophage M1/M2 polarization. Eur. J. Pharmacol..

[19] Homem C.C., Repic M., Knoblich J.A. (2015). Proliferation control in neural stem and progenitor cells. Nat. Rev. Neurosci..

[20] Schuster S.J., Svoboda J., Chong E.A. (2017). Chimeric antigen receptor T cells in refractory B-cell lymphomas. N. Engl. J. Med..

[21] Doran A.C., Yurdagul A., Tabas I. (2020). Efferocytosis in health and disease. Nat. Rev. Immunol..

[22] Mosca M., Tani C., Vagnani S., Carli L., Bombardieri S. (2014). The diagnosis and classification of undifferentiated connective tissue diseases. J. Autoimmun..

[23] Fasano A., Visanji N.P., Liu L.W., Lang A.E., Pfeiffer R.F. (2015). Gastrointestinal dysfunction in Parkinson's disease. Lancet Neurol..

[24] Dalakas M.C. (2015). Inflammatory muscle diseases. N. Engl. J. Med..

[25] Racanelli A.C., Kikkers S.A., Choi A.M., Cloonan S.M. (2018). Autophagy and inflammation in chronic respiratory disease. Autophagy.

[26] Aveyard P., Gao M., Lindson N. (2021). Association between pre-existing respiratory disease and its treatment, and severe COVID-19: a population cohort study. Lancet Respir. Med..

[27] Kang D., Pikhitsa P.V., Choi Y.W. (2014). Ultrasensitive mechanical crack-based sensor inspired by the spider sensory system. Nature.

[28] Syrjänen S., Syrjänen K. (2021). HPV-associated benign squamous cell papillomas in the upper aero-digestive tract and their malignant potential. Viruses.

[29] Jacobs C.G., Spaink H.P., van der Zee M. (2014). The extraembryonic serosa is a frontier epithelium providing the insect egg with a full-range innate immune response. Elife.

[30] Kuper C.F., Pieters R.H., van Bilsen J.H. (2021). Nanomaterials and the serosal immune system in the thoracic and peritoneal cavities. Int. J. Mol. Sci..

[31] Foxman B. (2010). The epidemiology of urinary tract infection. Nat. Rev. Urol..

[32] Ogawa Y., Kinoshita M., Shimada S., Kawamura T. (2018). Zinc and skin disorders. Nutrients.

[33] McShane L., Tabas I., Lemke G. (2019). TAM receptors in cardiovascular disease. Cardiovasc. Res..

[34] Zhao J., Zhang W., Wu T. (2021). Efferocytosis in the central nervous system. Front. Cell Dev. Biol..

[35] Davra V., Kumar S., Geng K. (2021 Feb 1). Axl and Mertk receptors cooperate to promote breast cancer progression by combined oncogenic signaling and evasion of host antitumor immunity. Cancer Res..

[36] Cabezón R., Carrera-Silva E.A., Flórez-Grau G. (2015 Apr). MERTK as negative regulator of human T cell activation. J. Leukoc. Biol..

[37] Peeters M.J., Dulkeviciute D., Draghi A. (2019). MERTK acts as a costimulatory receptor on human CD8^+^ T cells. Cancer Immunol. Res..

[38] Lindsay R.S., Whitesell J.C., Dew K.E. (2021). MERTK on mononuclear phagocytes regulates T cell antigen recognition at autoimmune and tumor sites. J. Exp. Med..

[39] Giroud P., Renaudineau S., Gudefin L. (2020). Expression of TAM-R in human immune cells and unique regulatory function of Mertk in IL-10 production by tolerogenic DC. Front. Immunol..

[40] Liu M., O'Connor R.S., Trefely S. (2019). Metabolic rewiring of macrophages by CpG potentiates clearance of cancer cells and overcomes tumor-expressed CD47− mediated ‘don’t-eat-me’signal. Nat. Immunol..

